# Clinical Performance of a Salivary Amylase Activity Monitor During Hemodialysis Treatment

**DOI:** 10.4137/bmi.s997

**Published:** 2008-09-15

**Authors:** Masaru Shimazaki, Takayuki Matsuki, Kazuaki Yamauchi, Michihiro Iwata, Hiroshi Takahashi, Kenichi Sakamoto, Junichi Ohata, Yuichi Nakamura, Yusuke Okazaki

**Affiliations:** 1 Division of Hemodialysis, Shin-Nittetsu Muroran General Hospital, Muroran, Japan; 2 Department of Cardiology, Shin-Nittetsu Muroran General Hospital, Muroran, Japan; 3 Department of Internal Medicine, Shin-Nittetsu Muroran General Hospital, Muroran, Japan

**Keywords:** biomarker, chronic renal failure, hand-held monitor, hemodialysis, salivary amylase activity, stress

## Abstract

The hemodialysis procedure is thought to be a physical stressor in the majority of hemodialyzed patients. Previous studies suggest that elevated salivary amylase level may correlate with increased plasma norepinephrine level under psychological and physical stress conditions. In this study, we investigated biological stress reactivity during hemodialysis treatment using salivary amylase activity as a biomarker. Seven patients (male/female = 5/2, age: 67.7+/−5.9 years) who had been receiving regular 4 h hemodialysis were recruited. Salivary amylase activity was measured using a portable analyzer every hour during the hemodialysis session. Salivary amylase activity was shown to be relatively stable and constant throughout hemodialysis, whereas there were significant changes in systolic blood pressure and pulse rate associated with blood volume reduction. Our results show that hemodialysis treatment per se dose not affect salivary amylase activity.

Since stress reaction is a biological reaction caused by stress-induced alterations in the autonomic nervous system and endocrinological and immunological functioning, the complexity of this relationship has not been fully elucidated. It is of interest to let us know how a person feels own condition somehow or other. There have been many attempts to quantify stress by various methods, including psychological tests and measurements of hormonal, cardiovascular responses and other physiological parameters. On really quantifying stress, it is a delicate problem to apply which indicator can do better that in a lot of indicators associated with stress. There are several indicators that are associated with stress and can be measured easily and economically. Recently, a portable salivary amylase activity analyzer has become available (Yamaguchi et al. 2004). Because salivary amylase is innerved by the sympathetic nervous system, it is expected that change in salivary amylase activity correlates well with stress (Chatterton et al. 1996). It has been suggested that salivary amylase activity is a useful index of plasma norepinephrine levels under a variety of stressful conditions, since it appears that increased sympathetic nervous activity is a major stimulator of amylase secretion (Skosnik et al. 2000). Not only psychological (Chatterton et al. 1997; Nater et al. 2006) but also physical stressors (Nexo et al. 1988; Steerenberg et al. 1997; Walsh et al. 1999; Skosnik et al. 2000) are able to stimulate salivary amylase activity. Saliva sampling is a noninvasive, painless, and convenient test modality.

Since first successfully performed in 1945, hemodialysis (HD) has become a routine therapeutic procedure (Kolff, 2002). However, despite significant improvements in HD equipment and improvement in patient monitoring, acute complications can still occur during the therapy. Intradialytic hypotension requiring medical intervention occurs in 10% to 30% of HD treatments (Levin et al. 1990). During volume removal by ultrafiltration, a reduced plasma refilling rate coupled with impaired compensatory physiologic responses to hypovolemia may contribute to the pathogenesis of intradialytic hypotension (Liangos et al. 2005). On-line blood volume monitoring techniques have been used to control intradialytic hypotensive episodes, but their effectiveness is controversial. Although occasionally asymptomatic, patients with hypotension may suffer from various symptoms including light-headedness, muscle cramps and nausea. Besides, HD patients must also endure the pain from stabs with injection needles in HD sessions. The HD procedure is thought to be a physical stressor in the majority of hemodialyzed patient. We speculated that the HD procedure is a stressor and leads to enhanced salivary amylase activity with progress of the procedure. Thus, in the present study, we investigated the biological stress reactivity using salivary amylase activity as a biomarker. A portable salivary amylase activity analyzer was tested during the HD session.

## Materials and Methods

Outpatients with stable disease conditions who had been treated with ordinary chronic regular HD for 1 year or longer were enrolled in this study. Only outpatients aged 55 years or more were recruited for this study because there might be a difference in the reactiveness of salivary amylase activity in young subjects and aged subjects (Meyer et al. 1940; Arglebe, 1979; Zhang, 1984; Ben-Aryeh, 1986; Salvolini, 1999). All of the patients were dialyzed three times a week for 4 h each time. We excluded patients treated with any drug that may have contributed to action of the sympathetic nervous system, such as alpha blockers, beta blockers (Nederfors et al. 1994; van Stegeren et al. 2006), alpha-methyldopa, etilefrine hydrochloride, amezinium metilsulfate, midodrine hydrochloride, L-threo-3, 4-dihidroxyphenylserine, and antide-pressants (Koller et al. 2001). The study was conducted in accordance with the declaration of Helsinki. A full explanation of the study, focusing on the purpose of the study and the precise procedures, was given to all of the patients before enrollment. Informed consent to participate in this study was obtained from each patient.

Salivary amylase activity was measured using a hand-held salivary amylase monitor manufactured by Nipro (Osaka, Japan). This analyzer enables automatic measurement of salivary amylase activity, using a dry-chemical system, within 1 min from collection to completion of the measurement (Yamaguchi et al. 2004; Yamaguchi et al. 2006b). The tip of the testing strip was soaked in a buffer containing Gal-G2-CNP (2-chloro-4-nitrophenyl-4-O-beta-D-galactopyranosylmaltoside), which acts as a substrate, and chromogen and was then dried. The definition of one unit activity (U) per mass of enzyme (Yamaguchi et al. 2004) is that this activity produces 1 micro mol of the reducing sugar maltose in 1 min. The tip of the testing strip was set under the tongue for 30 sec to collect saliva. Then, the testing strip was immediated inserted into the analyzer, and the result was displayed automatically. The first sample was measured as baseline salivary amylase activity, before vein cannulation. After vein cannulation, additional samples were obtained at 1-h intervals throughout the HD session, until the needle was removed after completion of HD. Subjects lay on a bed when samples were collected. Continuous monitoring of % blood volume reduction was performed using CRIT-LINE III (Hemametrics, Salt Lake City, UT, U.S.A.) during the HD session. Since it has been suggested that amylase activity have a diurnal behavior (Artino et al. 1998; Nater et al. 2007), the HD session was performed between 9:00 and 13:00 h, during which time patients did not have lunch.

Changes in salivary amylase activity, systolic blood pressure, pulse rate, and % blood volume reduction were analyzed using one-way repeated measures analysis of variance. Statistical significance was defined at the level of p < 0.05. Data are given as means and standard deviations. In figures, box and whisker plots indicate median value with interquartile range and 10%–90% range in the panel. Since salivary amylase activity showed variations in individuals, all of the measured amylase activity values were converted into logarithmic values (Yamaguchi et al. 2006a).

## Results

**Figure 1:**
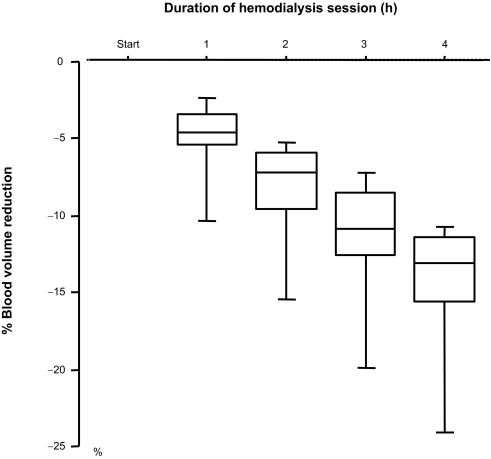
Change in % blood volume reduction during hemodialysis. Blood volume significantly (p < 0.0001) decreased steadily throughout hemodialysis. Box and whisker plots indicate median value with interquartile range and 10%–90% range.

The participants in this study included 5 men and 2 women. The mean age of the participants was 67.7 (SD = 5.9, range 58–76) years, and the mean duration of dialysis was 84.0 (SD = 59.1, range 14–188) months. Their primary renal diseases were chronic glomerulonephritis in 5 cases and diabetic nephropathy in 2 cases. The mean value of salivary amylase activity at the baseline condition was 17.7 (SD = 17.4, range 2–49) kU/L. The mean ultrafiltration volume (removal of fluid) during HD was 2.4 (SD = 0.7, range 1.1–3.1) L. Blood volume decreased steadily throughout the HD session (p < 0.0001) (Fig. 1).

**Figure 2:**
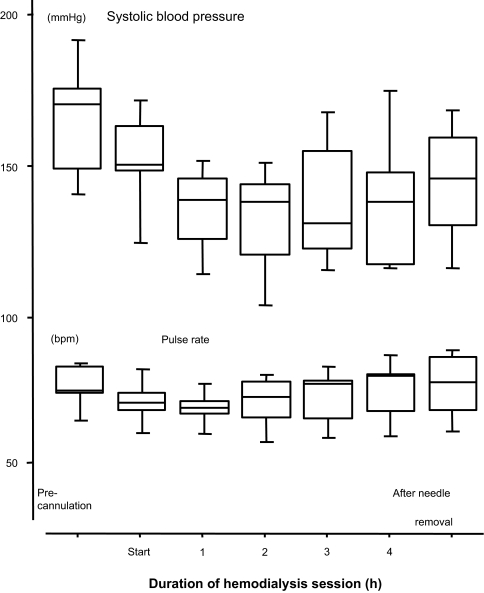
Changes in systolic blood pressure and pulse rate during hemodialysis. A significant decrease in systolic blood pressure (p = 0.0011) with a trough at 2 h after the start of hemodialysis was observed. Pulse rate significantly (p = 0.0003) decreased for the first hour after the start of hemodialysis and thereafter continued to increase gradually. Box and whisker plots indicate median value with interquartile range and 10%–90% range.

Time courses of systolic blood pressure and pulse rate showed significant patterns of change, as can be seen in Figure 2. HD resulted in a significant decrease in systolic blood pressure with a trough at 2 h after the start of HD (p = 0.0011). Pulse rate decreased for the first hour after the start of HD and thereafter continued to increase gradually (p = 0.0003).

**Figure 3:**
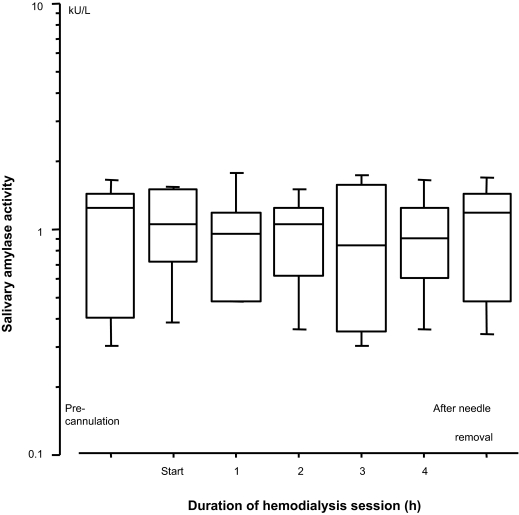
Change in salivary amylase activity during hemodialysis. Measured values were converted into logarithmic values. No significant effect associated with salivary amylase activity throughout hemodialysis was observed. Box and whisker plots indicate median value with interquartile range and 10%–90% range.

From the pre-cannulation to the completion of HD, salivary amylase activity was shown to be relatively constant and was not significantly different (Fig. 3). None of the patients complained of symptoms such as light-headedness, muscle cramps, nausea, cold sweat, dyspnea, chest pain, and fainting.

## Discussion

Salivary amylase activity response is thought to be sensitive to psychological stressors, such as parachuting or skydiving anticipatory coping stress (Chatterton et al. 1997), passive watching of a gruesome video and psychosocial examination (Bosch et al. 2003; Rohleder et al. 2004), and to physical stressors, such as running, intensive exercise, and exposure to heat and cold (Chatterton et al. 1996). In a series of studies in which subjects were exposed to physical and psychological stressors, significant correlations between salivary amylase activity and plasma norepinephrine were found. Nater et al. (2006) reported that general salivary amylase responses were associated with sympathetic tones, but not associated with general salivary cortisol responses, during stress conditions in a psychosocial examination. Using these results as support for the validity of measuring salivary amylase instead of catecholamine, several studies in which salivary amylase activity was measured as an indicator for norepinephrine were performed (Chatterton et al. 1996; Chatterton et al. 1997; Skosnik et al. 2000; Nater et al. 2006). In this study, we examined the influence of the HD procedure on salivary amylase activity in patients on HD. While there were significant changes in systolic blood pressure and pulse rate associated with blood volume reduction during HD, salivary amylase activity showed no difference during HD. The patients in this study did not show excess intradialytic hypotension causing discomfort, although they showed significant falls in blood pressure. The occurrence of episodes of hypotension during HD treatments may contribute to stress reaction. However, symptomatic hypotension did not occur in the current study. Consequently, it was unlikely that an increase in salivary amylase activity would be observed during routine HD treatment. Chicharro et al. (1998) reported that a saliva threshold exists during physical exercise. Routine HD treatment might have an effect on low stress intensity below the saliva threshold level. As the patients had been undergoing HD for a long time, they might have become accustomed to stresses of the HD procedure.

Results of studies regarding the relationship between salivary amylase activity and age are discrepant: results of studies have shown that salivary amylase activity declines with aging (Meyer et al. 1940; Ben-Aryeh, 1986), that salivary amylase activity does not differ with aging (Arglebe, 1979; Salvolini, 1999), and that salivary amylase activity is increased in the elderly (Zhang, 1984). Also, it is of interest to consider the possible influence of age in dialysis patients. Although the subjects in our study were elderly patients, the basal level of salivary amylase activity was similar to that in a study by Yamaguchi et al. (2006a), who measured the basal salivary amylase activity in healthy young adults using a hand-held analyzer.

The results of our study should be interpreted within the inherent limitations of the study. Our patient population was relatively small. Aged patients were enrolled in the current study, but young patients were not recruited in consideration of the possible influence of age. The relationship between salivary amylase activity and age is discrepant. Moreover, to achieve blood pressure control, most patients on HD would require multiple antihypertensive agents, including alpha blocker, beta blocker and alpha-methyldopa, and sympathomimetic agents, including etilefrine hydrochloride, amezinium metilsulfate, midodrine hydrochloride and L-threo-3, 4-dihidroxyphenylserine, which might contribute to the action of the sympathetic nervous system. Thus, most of the patients in our hospital were excluded before enrollment by the exclusion criteria. If possible, a case-control study should be designed. However, our study was not conducted as a case-control study. It is considered ethically problematic to perform HD in healthy subjects as controls. Therefore, we could not perform such a study design. Therefore, our results might not be applicable to other medicated patients mentioned above.

In summary, our results show that the routine HD procedure per se does not affect salivary amylase activity in patients treated with chronic HD. However, changes in salivary amylase activity possibly reflect autonomic changes. Salivary amylase is thus part of a general psychobiological stress response, which makes salivary amylase a very interesting variable that can easily be obtained.
